# Recent Design Development in Molecular Imaging for Breast Cancer Detection Using Nanometer CMOS Based Sensors

**DOI:** 10.1155/2012/680262

**Published:** 2012-12-24

**Authors:** Dung C. Nguyen, Dongsheng (Brian) Ma, Janet M. W. Roveda

**Affiliations:** ^1^BIO 5 Institute, Department of Electrical and Computer Engineering, University of Arizona, Tucson, AZ 85721, USA; ^2^Department of Electrical and Computer Engineering, The University of Texas at Dallas, 800 W Campbell Road, EC33, Richardson, TX 75080-3021, USA

## Abstract

As one of the key clinical imaging methods, the computed X-ray tomography can be further improved using new nanometer CMOS sensors. This will enhance the current technique's ability in terms of cancer detection size, position, and detection accuracy on the anatomical structures. The current paper reviewed designs of SOI-based CMOS sensors and their architectural design in mammography systems. Based on the existing experimental results, using the SOI technology can provide a low-noise (SNR around 87.8 db) and high-gain (30 v/v) CMOS imager. It is also expected that, together with the fast data acquisition designs, the new type of imagers may play important roles in the near-future high-dimensional images in additional to today's 2D imagers.

## 1. Introduction

Today, the mainstay of clinical imaging still vastly depends on traditional anatomical imaging methods such as ultrasound (US), computed X-ray tomography (CT), and magnetic resonance imaging (MRI). With the uprising genomic and proteomic technologies, cancer detection is no longer just detecting tumor position and size, it also involves more precise understanding of anatomical structures of cells that would lead to different treatment in managing cancer clinically. The center of this new transformation is molecular imaging, a new technique that allows one to combine molecular information with physiological study. It is believed by many researchers that this new development will detect molecular alterations related to cancer, will help evaluate existing cancer treatment in real time, and will eventually reshape the cancer medicine delivering process in the near future [1–3]. Here, “real-time” refers to treatment followups, information sharing, and collaborations among physicians and patients at the same time. Two key trends of molecular imaging techniques include magnetic resonance imaging (MRI) and optical imaging. However, high cost and possible invasive penetrations are the major road blocks. For example, it has been estimated that the MRI may cost each patient from $2000 up to $3800 for each visit [4], while optical probes require prescreening using traditional anatomical imaging approaches to first locate problematic areas and then penetrate through skin/muscles to the questionable locations. Such techniques would not be suitable for real-time tracking of cancer development and detection. 

Over the last several years, a lot of effort has been focused on how to improve the mainstream techniques to improve cancer detection rate without increasing the cost and additional biopsy-type penetrations. One trend is to improve the existing X-ray techniques. In this paper, we intend to review our recent progress in X-ray-based breast cancer detection system design. We focus on the device, the circuit, and the embedded software and hardware perspectives to reveal the potential of the future mammographic system design considering the new development in the nanometer CMOS technologies. 

It is worth mentioning that the current film screen-based mammography is very mature and successful. Yet this popular technique still misses as many as 20% of cancer cases [5] due to the anatomical noise. This type of noise is caused by the overlap of normal structures within breast tissues. Very common in a standard two-dimensional mammogram is that tissue structures tend to superimpose on each other. One approach to reduce the anatomical noise is tomosynthesis. Researchers [6, 7] have reported that tomosynthesis may increase cancer detection rate by 16% and decrease false positive cases by 85% compared with the film-based mammography. However, the design of such a system to support tomosynthesis is tricky. This is because tomosynthesis algorithm requires multiple images of the imaged breast. Thus, it also demands multiple image acquisitions. On the other side, we would like to limit to a comparable amount of radiation dose. That is, if we wish to read out 10 to 15 images, each image should only use 1/10 to 1/15 of the single image radiation dose without tomosynthesis algorithm. However, such a low X-ray dose would lead to high level of noise in the current mammographic designs. How to design a system that overcomes these contradicting requirements is the main focus of this paper. 

For the rest of this article, we first discuss the potential of Silicon-On-Insulator device for the new pixel sensor and its design. Then, we introduce the current research in fast data acquisition and high dynamic range design and their contribution to the CMOS based molecular imager. Finally, we review the upcoming new generation of X-ray generators and their potentials in discovering pathological development of cancer cells. 

## 2. Nanometer CMOS-Based Molecular Imaging with Silicon on Insulator

Together with Bioptics Inc., we were among the first ones that designed the architecture using Silicon-On-Insulator (SOI) and the back-side illumination etched substrate. This new architecture makes it possible to increase the fill factor (up to 100%) and to separate the photodiodes from the rest of the device electronics to reduce coupling noises [8]. 

**Figure 1:**
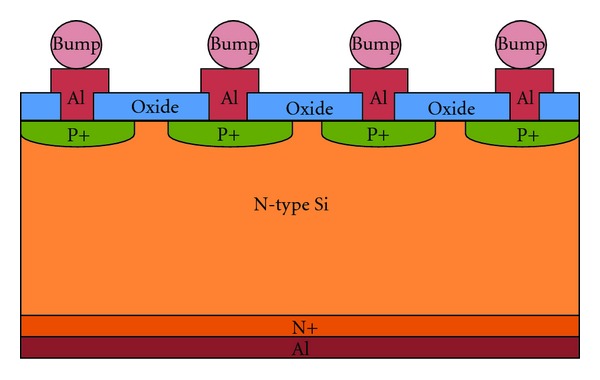
The cross-section of the newly proposed device and its design [7].

**Figure 2:**
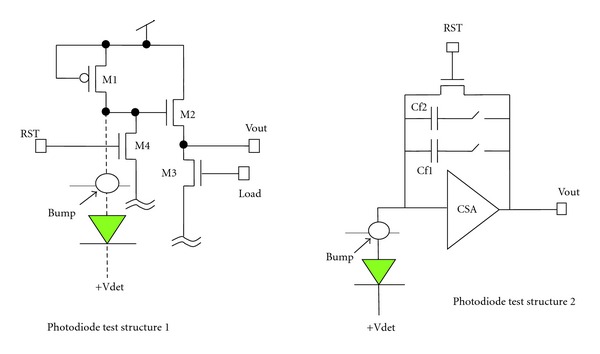
The test circuits of the proposed SOI sensors [7].

**Figure 3:**
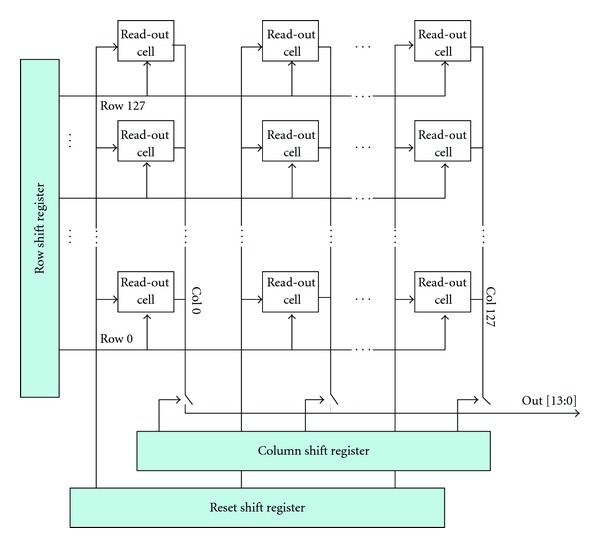
The test architecture for the pixel array [8].

The standard bulk CMOS technology-based imager faces two roadblocks: the short depth of n-well and the high parasitic capacitance. It is well known that high-resolution requirement demands long exposure time. The shorter the well, the less the exposure time we can have. Together with the impact from parasitic capacitance, the speed and performance of the CMOS imager are both limited. One advantage of the SOI technology is its low parasitic capacitance which leads to high speed and the potential to extract images with less exposure time. Currently, the main-stream SOI techniques include two types of technologies: fully depleted SOI (FD-SOI) and partially depleted SOI (PD-SOI), while in the manufacture process, PD-SOI is less expensive but suffers from kink effect (different slope rate in the saturation region in the *I*-*V* curve) which will affect the SNR ratio of the imager [9]. This is the reason why we choose FD-SOI instead of PD-SOI.  Figure 1 demonstrates the device cross-section. Figures 2 and 3 display the design architecture and the test structure of the new SOI-based molecular imager, respectively.

To fully utilize the advantage of SOI technique, we also developed a new hybrid architecture to design sensors and its auxiliary circuitry. This is different from the monolithic architecture where we need to integrate detectors and read-out circuitry in a high-resistivity silicon substrate [10]. 

**Figure 4:**
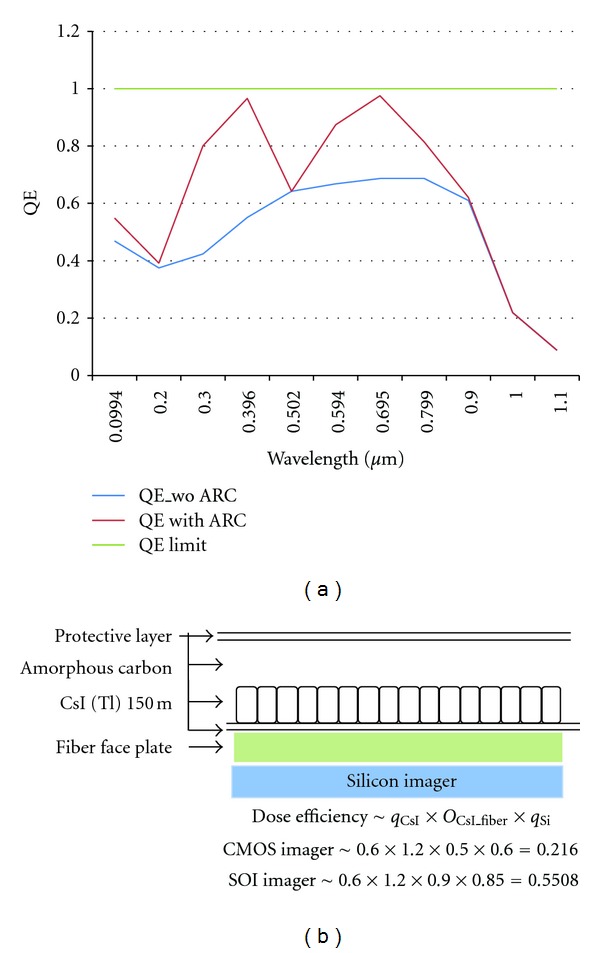
(a) DOE simulation results for structure 1 and structure 2 in Figure 2 at different optical wavelengths. (b) The cross-section structure of optical architecture.

In general, this requires two-sided wafer process. In the hybrid pixel architecture, the read-out integrated circuit connects with the detector material by solder bumps [11, 12]. Compared with the monolithic technique, the hybrid technology is able to integrate different detector platforms with the same read-out circuitry. The hybrid technique also reduces the design cost as it simplifies the design process of the read-out circuitry which can be optimized independently from the detector. The test architecture (Figure 3) includes a charge sensitive amplifier (CSA), a discriminator, a sigma-delta analog digital converter [13], and a 14-bit counter/shift register. The last two test modules are a complete 128 × 128 photon counting array and a 128 × 128 in pixel analog-to-digital array [8].  Table 1 recorded the read-out noises for the 128 × 128 array in Figure 3. Two different ways to store the output signals of photons include counting approach and the integrating method. The later one sums up all the input signals including noise which causes low signal-to-noise ratio, short dynamic range, and a redistribution of photons with different energy level. Photons of higher energy level store more charges in the detector and will generate a higher voltage or current. Note that even though the higher-energy-level X-rays can pass through the patient breast easily, they do not necessarily carry more useful information than the lower-energy ones. On the other hand, these higher-energy ones may also defer photon integration time. In the photon counting method, the input signal height from a photon is compared to an energy window threshold set in a comparator. If the signal is within the energy window threshold range, the value of a counter is incremented. Each photon within this window threshold has the same value. Thus, the photon counting approach has the ability to set an appropriate threshold to remove the background noise, which in turn improves the signal-to-noise ratio and the dynamic range [8, 14]. Preliminary Silvaco/Atlas P-i-N photodiode Detective Quantum Efficiency (DQE) simulation results at 50 V is in Figure 4(a). The estimate dose efficiency for the proposed device can be found in Figure 4(b), where *q*_CsI_ = photon absorption efficiency of CsI(Tl) at 30 keV, *g*_CsI_ = the CsI(Tl) light gain, *O*_CsI_fiber_ = the optical coupling between CsI(Tl) and the fiber faceplate, and *q*_Si_ = the silicon quantum efficiency.

## 3. 3D Imaging versus 2D Imaging to Improve Diagnostic Rate 

One effective way to avoid anatomical noises is by using higher-dimensional approaches, that is, 3D and 4D breast computed tomography (Breast CT) [15] and tomosynthesis. Still, the major challenge is the dramatic increase in the data volume: for 3D tomosynthesis, 15 frames of images are required comparing with 2 frames for the traditional 2D ones. This is equivalent to a 7x increase in the data volume (i.e., for cone-beam CT; this is about 300 projections) [16]. In addition, there will be an increase in the X-ray dose as well. For the “average” breast (compressed thickness of 5.0 cm and a 50% glandular fraction), a digital breast tomosynthesis (DBT) acquisition results in an 8% increase in the X-ray dose comparing with 2D ones. For high-density breasts, the increased amount can be as high as 83% [17, 18].

Over the past several years, great efforts have been invested into analog-to-information converter (AIC) [19] to acquire raw sample at a low rate while accurately reconstructing the compressed signals. The key components under investigations were analog-to-digital converters, random filtering and demodulations. Authors from [20] were the first ones that applied compressive sensing into pixel array data acquisition systems. Instead of directly extracting pixel array data to column buffers and then processing with A/D converters, the authors used random selection measurement matrix to regroup the pixel data. And then, the data are fed into A/D at a much lower sample rate than the original design. By doing this, they implemented the random selection measurements and multipliers using analog components. Such designs choose “heavy” analog front end to reduce the sample rate and data amount at A/D. For example, in the traditional signal/data flow, the A/D converter is placed right after pixel array. That is, the pixel data are directly digitized at the Nyquist sample rate with compressive sensing where the A/D converter is placed after random selection/demodulation. The sample rate is a lot lower. Even though compressive sensing algorithms helped us to reduce the sample rate of A/D converter, it comes with a price. It requires analog front end to achieve randomized measurements which in turn lead to large analog computing units at the front end. These components are cumbersome and slow. For example, an analog multiplier works at 10 MHz with over 200 ns set-up time. Not to mention that most elements in the front end use 0.25 um technology node, some exploit 0.5 um technology node (i.e., floating gate technology to store random selection coefficients). While compressive sensing algorithms provide a promising future to reduce data, how to effectively implement such a scheme on hardware is still an open question.

High-dynamic-range imaging is an emerging field that has the potential to cause a great scientific and technological impact in the near future. In this paper, we aim at covering the field of high-dynamic-range imaging and its applications in extending the imager dynamic range and the impact on X-ray dose reduction for medical imaging application. A typical pixel cycle in an integrating-type image sensor receiving a constant light intensity is shown in Figure 3. An initial reset cycle zeroes the intensity signal, which later increases linearly throughout the integration time. The illumination signal is thus limited by fabrication process parameters, system factors, and circuit implementation regardless of the type of pixel used. Additionally, the noise of the pixel read-out circuitry and other noise sources overwhelm the illumination signal in low light situations. The ratio between the illumination that saturates the pixel and the minimum detectable illumination is the dynamic range (DR) of the image sensor. Low-noise circuitry, careful engineering of the photodiode reverse saturation current, and other improvements can help lower the noise floor, and has been previously attempted by several techniques. Previous efforts to increase the DR of a CMOS imager have introduced various image-acquiring schemes including logarithmic, a combination of linear and logarithmic, well-capacitance adjusting, dual or multiple sampling, and multiple integrations [21–23].

## 4. Conclusion and Future Work

Based on our experience [8, 24] and existing research works [5, 7, 17, 25], it is apparent that the new technologies in CMOS image sensor designs have great potential in improving the current mammographic breast cancer detection. To be more specific, using SOI technology can provide low-noise (SNR around 87.8 db) and high-gain (30 v/v) CMOS imager. This type of imagers together with fast data acquisition designs may play important roles in the near future high-dimensional images. It is also worth mentioning the new X-ray-free electron laser technique [25–27]. Comparing with the molecular imager reviewed in the paper (we can have images that reveal cells of size of tens of micro-meters), this new technique can take a snapshot of molecule size object in femtosecond (fs). This is the first time we can monitor proteins and chromosomes in real time. It is believed that this new technique can reveal the relationship of genomic and proteomic technologies and cancer growth. Its promises to the breast cancer detection yet needs to be further confirmed and calls for strong collaborations among scientists and researchers from different disciplines including radiology, pathology, device designers, and circuit and system designers.

## Figures and Tables

**Table 1:** Read-out noise estimation (worst case).

Simulate read-out noise	Description	Value	Unit
Electronic gain		29.6	electron/ADU
Dark rate	30.6 ADU/sec ∗29.6 electron/ADU	906	electron/pixel/sec
Dark current noise	sqrt(dark rate ∗*t*)		
Read-out noise	Gain × std_dev(offset)/sqrt(2)		
	29.6 ∗ 3.27/1.414	69	electron/pixel (for 500 kHz rate)
SNR	Full well potential/read-out noise		
	20∗ log10(1700000/69)	87.8	dB
